# Development of a Purple-Leaf Perillene Chemotype Line in *Perilla frutescens* Reveals Incomplete Linkage with Leaf Color

**DOI:** 10.3390/plants15071044

**Published:** 2026-03-28

**Authors:** Wei Wei, Bin Wang, Zhaoyuan Li, Yang Liu, Hongliang Ji, Zhou Wu, Guangyao Ma, Yuxuan Sun, Tiantian Zhang, Yanbing Liu, Longfeng Feng, Yue Jin, Tingting Wang, Qiuling Wang, Zhihui Gao, Jianhe Wei

**Affiliations:** 1 Key Laboratory of Bioactive Substances and Resources Utilization of Chinese Herbal Medicine, State Key Laboratory of Bioactive Substance and Function of Natural Medicines, Institute of Medicinal Plant Development, Chinese Academy of Medical Sciences & Peking Union Medical College, Beijing 100193, China; 2 Hainan Provincial Key Laboratory of Resources Conservation and Development of Southern Medicine, Hainan Branch of the Institute of Medicinal Plant Development, Chinese Academy of Medical Sciences & Peking Union Medical College, Haikou 570311, China

**Keywords:** *Perilla frutescens*, chemotype, perillene, perillaldehyde, leaf color, incomplete linkage

## Abstract

*Perilla frutescens*(L.) Britt. (*P. frutescens*) is an important medicinal and aromatic plant, whose leaf color and chemotype strongly influence its medicinal quality and economic value. All the previously discovered perillene (PL)-type *P. frutescens* are double-sided green, and whether the PL-type trait is tightly linked with the green-leaf trait in genetics remains to be clarified. This study aimed to address this question and attempt to create purple-leaf PL-type germplasm through perillaldehyde (PA) × PL hybridization. Three parallel experiments were conducted using purple-leaf PA-type *P. frutescens* as male parents and green-leaf PL-type *P. frutescens* as female parents. Chemotypes were identified by gas chromatography (GC). Association analyses between leaf color and chemotype were performed in segregating F_2_ populations. Genes involved in leaf color formation and PL biosynthesis were mapped onto the published Hoko-3 reference genome to provide genomic evidence for the genetic relationship between the two traits. All F_1_ individuals were uniformly PA-type. The three F_2_ populations exhibited distinct leaf color–chemotype association patterns: Z01 (*n* = 118) showed a strong association (Fisher’s exact *p* = 9.13 × 10^−10^; φ = 0.564), Z02 (*n* = 117) showed no detectable association (*p* = 0.9; φ = 0.012), and Z03 (*n* = 88) showed a moderate association (*p* = 0.00669; φ = 0.289). Importantly, purple-leaf PL-type recombinants were obtained in F_2_ populations and stably maintained through subsequent generations (F_3_–F_5_), demonstrating that the PL-type trait is not tightly linked with the green-leaf trait in *P. frutescens.* Genomic mapping genes related to leaf color and PL biosynthesis are distributed across multiple chromosomes and usually present as multiple loci, which is consistent with the pattern of incomplete linkage. The PL-type trait is recessive and not genetically tightly linked to the green-leaf traits in *P. frutescens*. The successful creation of a purple-leaf PL-type germplasm breaks the historical phenotypic constraint and provides a novel material for further dissection of the molecular mechanisms regulating secondary metabolism and organ coloration in *P. frutescens*.

## 1. Introduction

*Perilla frutescens* (L.) Britt. (*P. frutescens*) is an important aromatic plant that is of both edible and medicinal use, widely distributed across Asia [1]. It is renowned for its unique volatile oil and its pharmacological activities [2]. Based on the relative abundance of the major volatile constituents, multiple chemotypes have been defined [2], such as types of perillaldehyde (PA), perillene (PL), perillaketone (PK), citral (C), etc. Among all chemotypes, PA and PL are particularly notable because of their distinct aroma properties and high utilization value in the pharmaceutical industry [3,4,5], especially the anti-inflammatory effect of PL. This makes the PL-type traits of *P. frutescens* a high-value target for breeding.

For the utilization of *P. frutescens*, chemotype and leaf color are both important traits. For example, the Chinese Pharmacopoeia 2020 edition requires that *P. frutescens* leaves be double-sided purple or single-sided purple while excluding double-sided green [2]. Perilla ketone (PK)-type green-leaf *P. frutescens* are used in Korea and Japan as a fresh vegetable, while the perillaldehyde (PA)-type *P. frutescens* with purple leaves are used as medicine. Moreover, according to the quality standards of essential oils of *P. frutescens* leaves in Huoxiang Zhengqi oral solution, PL content should not be less than 20% and PA content should not be less than 25% [2].

However, based on years of investigation by our research team, as well as a literature report [6], it was found that the natural population of the PL type is always green-leaf, and no purple-leaf PL type has been found, whereas both purple-leaf [6] and green-leaf varieties can be found in the natural population of PA-type plants in our previous study and other research. This obvious correlation raises a fundamental question of whether the trait of PL-type *P*. *frutescens* is tightly linked with green leaves in genetics. Clarifying this genetic relationship between the PL type and green leaves is crucial for understanding their regulatory mechanism. Furthermore, it is also indispensable for molecular breeding aimed at artificially constructing high-value PL-type *P*. *frutescens*.

Previous genetic studies on *P. frutescens* only focused on segregation among different chemotypes [7,8,9]. However, the direct evidence that supports a tight linkage between green leaves and the PL type is limited, and whether it represents a complete linkage remains to be clarified.

Until now, the genes in the biosynthetic pathway of PL have only been clarified partially, such as geraniol synthase (GS) [10] and geraniol dehydrogenase (GeDH) [11]. But the enzyme that catalyzes the last step of PL has not been revealed yet, which is speculated to be a cytochrome P450 [12]. The biosynthetic pathway of PA has been clarified. All genes in the PA biosynthetic pathway have been characterized, such as limonene synthase [13], limonene 7-hydroxylase CYP71D174 [14] and PA synthetase CYP71AT146 [15]. These identified genes, as well as the recently reported genomes of *P. frutescens* [16,17], provide the possibility of evidencing the linkage genomically.

In this study, we used hybrid populations derived from a cross between PA-type *P. frutescens* with purple leaves and PL-type *P. frutescens* with green leaves, tracking from F_2_ to F_5_ to clarify whether the PL type is tightly linked with the green color and explain the relationship at the genomic level by mapping all related genes of the two traits on the genome. This study is the first report of the creation of a new purple-leaf PL-type germplasm. It provides solid data support for the recessive inheritance of the PL type and green leaves and reveals the genetic linkage pattern between green leaves and the PL chemotype. Our research findings provide a new foundation for insights into the genetic mechanism of the formation of *P. frutescens* chemotypes.

## 2. Results

### 2.1. Parental Phenotypes and Chemotype Confirmation

**Figure 1:**
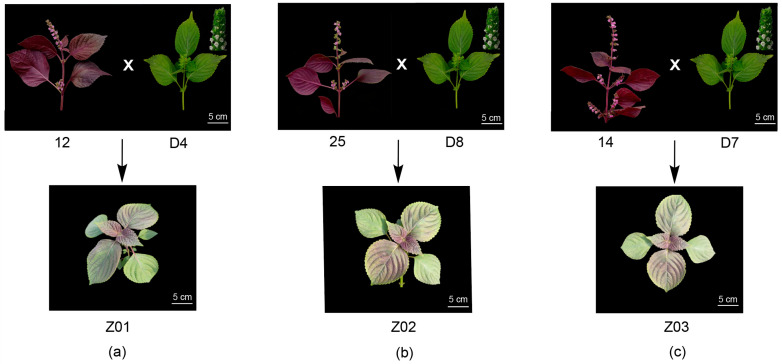
**Leaf** **colors of parental materials and F1 generation of** ***Perilla frutescens*****.** Phenotypes of the parental lines are shown: PA-type male parent is purple-leaf; PL-type female parent is green-leaf. Three independent crosses: (**a**) Z01: D4 (female, PL type) × 12 (male, PA type), (**b**) Z02: D8 (female, PL type) × 25 (male, PA type), and (**c**) Z03: D7 (female, PL type) × 14 (male, PA type). F1 generation individuals are all PA-type.

The parental lines used for crossing exhibited clearly distinct and stable leaf colors and chemotypes under uniform cultivation conditions. *P. frutescens* plants with purple leaves, named as 12, 25 and 14 (Figure 1), were identified as the PA type by GC-FID (Supplementary Figure S1, Tables S1 and S2). Green-leaf *P. frutescens* plants named as D4, D8, and D7 (Figure 1) were identified as the PL type by GC-FID (Supplementary Figure S2, Tables S3 and S4).

### 2.2. Inheritance Pattern of Chemotypes in F_1_ and F_2_ Generations

F_1_ plants of three independent crosses (Z01–Z03) displayed a uniform phenotype with intermediate mixed colors of green and purple (Figure 1). GC-FID analysis showed that all six F_1_ individuals were the PA type (Supplementary Table S5). It indicated that the PA type is dominant compared to the PL type. These results verify the successful hybridization between the two parental lines with different chemotypes and provide a reliable foundation for analyzing chemotype segregation and its relationship with leaf color in subsequent generations.

In the F_2_ generation, three independent crosses (Z01–Z03) generated a total of 323 individuals (Table 1). Among all F_2_ individuals, 250 were identified as PA-type and 73 as PL-type, corresponding to 77.4% and 22.6%, respectively (Table 1). The segregation result of chemotypes in F_2_ progeny of PA-type and PL-type *P. frutescens* further confirms the recessive inheritance of the PL type and green leaves.

### 2.3. Analysis of Genetic Linkage

**Figure 2:**
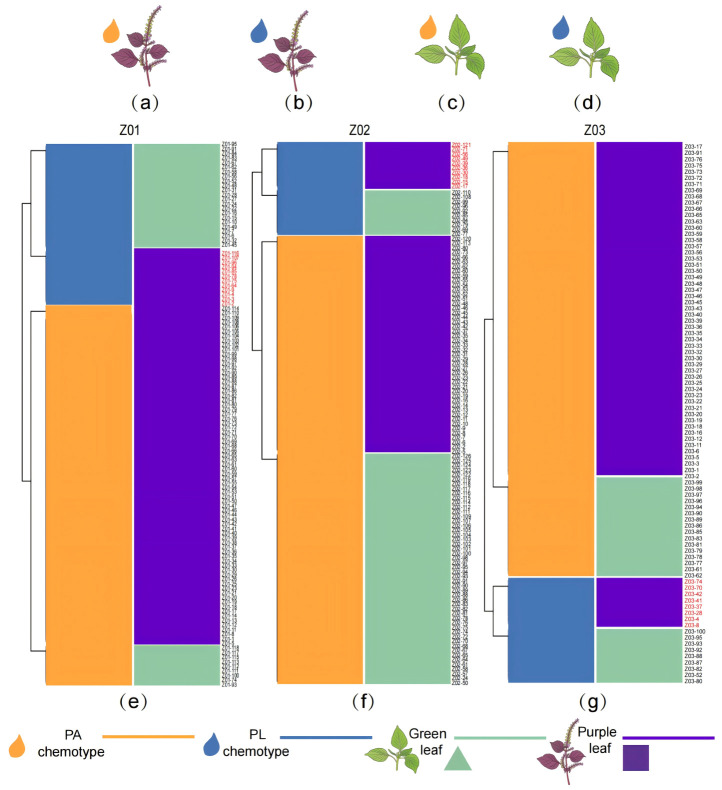
**Leaf color and chemotype segregation in F_2_ populations.** Leaf color and chemotype segregation in F_2_ populations: (**a**) PA-type purple-leaf *P. frutescens*. (**b**) PL- type purple-leaf *P. frutescens* (**c**) PA-type green-leaf *P. frutescens*. (**d**) PL-type green-leaf *P. frutescens.* (**e**) Leaf color and chemotype of each individual plant in F_2_ of Z01 populations. (**f**) Leaf color and chemotype of each individual plant in F_2_ of Z02 populations. (**g**) Leaf color and chemotype of each individual plant in F_2_ of Z03 populations. Orange color represents PA type, blue color represents PL type. Green color triangle represents green leaves. Purple color square represents purple leaves. Source data of chemotype identification of individuals of these three different F_2_ populations were listed in Supplementary Tables S6–S8.

In the F_2_ generation, the three independent populations (Z01, Z02, and Z03) allowed evaluation of the leaf color and chemotype relationship across genetic backgrounds. Each population was classified into four phenotypes (purple-leaf PA-type, purple-leaf PL-type, green-leaf PA-type, green-leaf PL-type) (Figure 2a–d). In all the three populations, individuals with all four different phenotypes were observed.

The degree of association and effect between leaf color and chemotype differed markedly among the three F_2_ populations. In Z01 (*n* = 118), the distribution deviated strongly from independence (χ^2^ (1) = 37.50, *p* = 9.13 × 10^−10^), with a large effect size (φ = 0.564). In contrast, Z02 (*n* = 117) showed essentially no association (χ^2^ (1) = 0.016, *p* = 0.900; φ = 0.012). Z03 (*n* = 88) displayed a significant but weaker association (χ^2^ (1) = 7.35, *p* = 0.00669; φ = 0.289) than the Z01 cross. These results indicate that the leaf color and chemotype association is not uniform across F_2_ populations derived from different parental individuals.

To unravel the genetic relationship between green leaves and the PL type, the recombinant phenotypes were defined firstly as green-leaf PA type and purple-leaf PL type. Recombinants were detected in all three F_2_ populations, demonstrating incomplete co-segregation between leaf color and chemotype. Specifically, recombinants occurred at 17.8% in Z01 (21/118; green-leaf PA-type = 9, purple-leaf PL-type = 12), 51.3% in Z02 (60/117; green-leaf PA-type = 50, purple-leaf PL-type = 10), and 27.3% in Z03 (24/88; green-leaf PA-type = 16, purple-leaf PL-type = 8) (Figure 2; Table 2).

### 2.4. Creation and Stability of Purple-Leaf PL-Type Germplasm

During the phenotype and chemotype screening of segregating progeny in the F_2_ generation, 12 plants were identified to be the purple-leaf PL type among a total of 118 individuals in the F_2_ population (8.8%). These phenotype results of F_2_ individuals provide direct evidence that there is not a tight linkage between traits of green-leaf and PL-type individuals.

**Figure 3:**
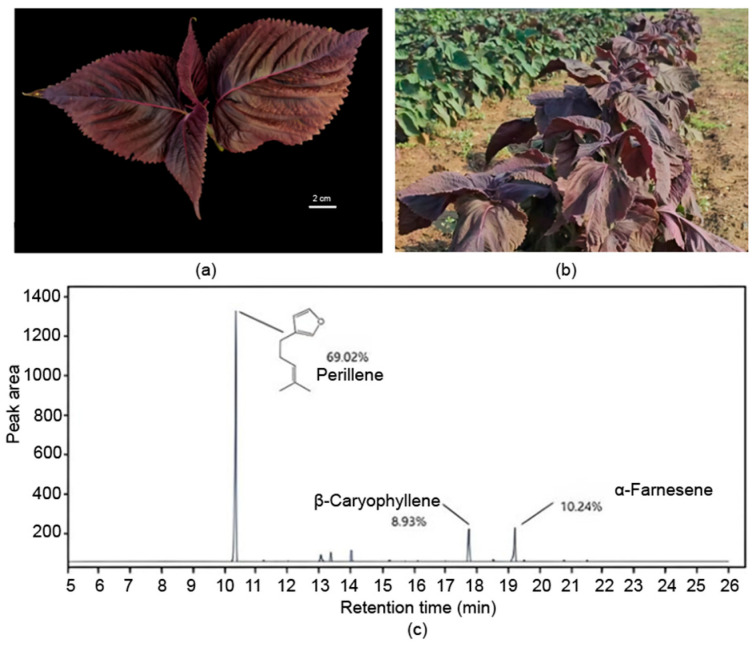
**Phenotype of representative PL-type individuals with purple leaf.** (**a**) Phenotype of purple-leaf PL-type individual. (**b**) Purple-leaf PL-type individual in F_4_ generation of Z01 population in the experimental field. (**c**) Gas chromatogram of purple-leaf PL-type individual.

These PL-type purple-leaf individuals displayed the same leaf color as that of the purple parental phenotype (Figure 3a), while their volatile oil chromatogram matched with the PL type (Figure 3c). The chromatographic patterns were similar to those of the green-leaf PL-type parent plants and clearly distinct from the purple-leaf PA-type parent plants (Supplementary Table S1). Together, these results confirm the successful creation of a purple-leaf PL-type *P. frutescens*.

**Figure 4:**
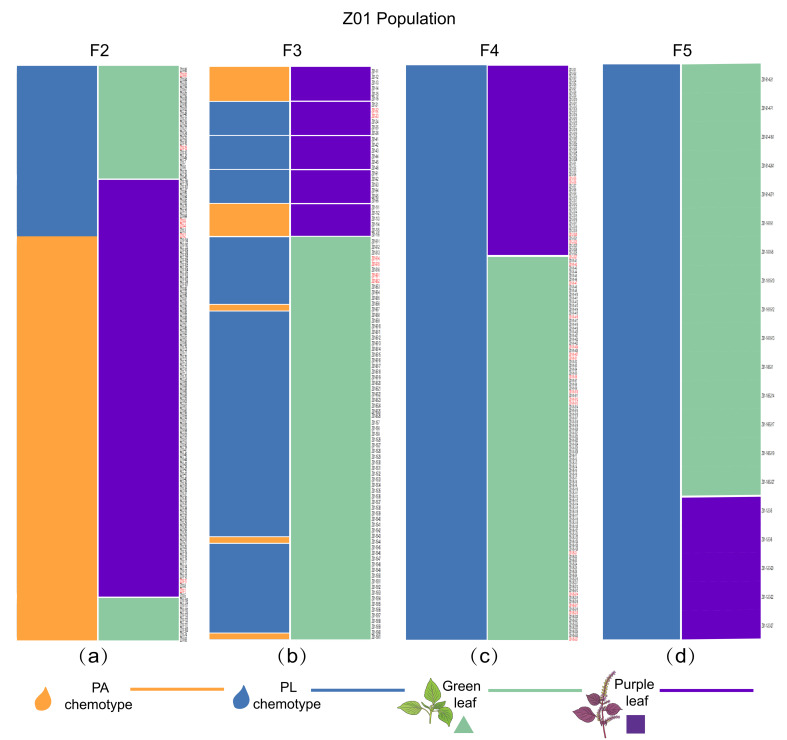
**Transmission of the purple-leaf PL-type*** **P. frutescens** ***in F_2_–F_5_ generations.** (**a**) Purple-leaf PL-type individuals in F_2_ population of the Z01 cross. (**b**) Purple-leaf PL-type individuals in F_3_ from the F_2_ population, which are highlighted in red in (**a**). (**c**) Purple-leaf PL-type individuals in F_4_ from the F_3_ population, which are highlighted in red in (**b**). (**d**) Purple-leaf PL-type individuals in F_5_ from the F_4_ population, which are highlighted in red in (**c**). Orange color represents PA type, blue color represents PL type. The green triangle represents green leaves. The purple square represents purple leaves. Source data of chemotype identification of individuals of plant in F_2_–F_5_ populations are listed in Supplementary Tables S6, S9–S11.

To determine whether the purple-leaf PL-type individual is heritable, we tracked the self-crossing generations of the purple-leaf PL type identified in the F_2_ generation. The leaf color of F_3_ progenies remained consistent with that of the corresponding F_2_ parent. The leaf color of F_4_ progenies remained consistent with that of the corresponding F_3_ parent, and the leaf color of F_5_ progenies remained consistent with that of the corresponding F_4_ parent. These results indicate that leaf color did not further segregate after F_3_ in the selected lineages. Importantly, GC-FID analyses confirmed that the PL type was retained in descendants across generations, demonstrating that purple-leaf PL-type individuals could be inherited through self-crossing rather than occurring occasionally or transiently (Figure 4). These stabilized purple-leaf PL-type *P. frutescens* materials constitute new germplasm resources for breeding programs of *P. frutescens*.

### 2.5. Genomic Distribution of Genes Involved in Anthocyanin Biosynthesis and Chemotype Formation

To explore the genomic context underlying the incomplete linkage of green leaves and the PL type, genes (Table 3) associated with anthocyanin biosynthesis, which controls leaf color, and genes identified in the biosynthesis pathways of different chemotypes, including the PL type, were mapped onto the published *Perilla frutescens* v. Hoko-3 reference genome [16]. Sequences of all genes curated from the literature were aligned to the Hoko-3 coding sequences, and only matches to the longest CDS with a sequence identity of >95% were retained. The chromosomal positions (Mb) of all retained loci are summarized (Supplementary Table S12).

**Figure 5:**
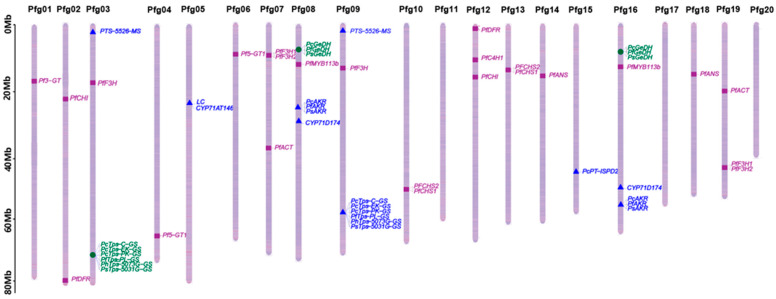
**Chromosomal distribution of genes of leaf color and chemotypes.** Purple squares represent genes in anthocyanin biosynthesis pathway. Green circles represent genes of PL biosynthetic pathway. Blue triangles represent genes in other chemotype biosynthetic pathway.

The results show that anthocyanin biosynthesis genes were distributed across multiple chromosomes, including loci on Pfg01, Pfg02, Pfg03, Pfg04, Pfg06, Pfg07, Pfg08, Pfg09, Pfg10, Pfg11, Pfg12, Pfg13, Pfg14, Pfg16, Pfg18, and Pfg19 (Figure 5). Several core pathway genes, such as chalcone synthase (*CHS*), chalcone isomerase (*CHI*), cinnamate-4-hydroxylase (*C4H*), dihydroflavonol reductase (*DFR*), anthocyanidin synthase *(ANS*), and flavonoid 3′-hydroxylase (*F3H*), were represented by more than one locus, consistent with the presence of paralogous copies in the reference genome (Figure 5). Key *MYB* transcription factors were also on more than one locus.

Overall, genes associated with leaf color and chemotype were dispersed across the genome and frequently represented by multiple loci, rather than being confined to a single shared chromosomal region.

## 3. Discussion

### 3.1. The Recessive Inheritance Evidence of PL-Type Plants

Previous experiments showed that chemotypes are genetically controlled [7,9,24] in *P. frutescens*. Since PL-type *P. frutescens* is rare, the genetic record for PA × PL chemotypes was blank [2]. Our results for F_1_ and F_2_ generations in Table 1 provide direct evidence of a recessive inheritance of the PL type. In addition, the recessive inheritance of the PL type with green leaves indicates that its occurrence requires a homozygous recessive genotype, which may explain why a purple-leaf PL type has not been reported before.

### 3.2. The Incomplete Linkage Between Leaf Color and Essential Oil Chemotype

Multiple resource survey studies of *P. frutescens* have reported an apparent correlation between leaf color and chemotype in *P. frutescens* [26,27]. Regarding whether the leaf color and essential oil chemotype traits are inherited independently, detailed evidence is missing. In this study, the segregation evidence revises the “tight linkage” assumption [1]. Since *P. frutescens* is an allotetraploid plant, the genetic control mechanism between the traits of green-leaf color and the PL type can be more complex. It is not just a simple genetic relationship of tight linkage. Instead, it is mostly consistent with incomplete linkage. The dispersed and multi-copy genomic distribution of genes controlling leaf color and chemotype supports a model of incomplete linkage between green leaves and the PL type. In addition, the creation of PL-type purple-leaf individuals and the results of the genomic map further support this conclusion, showing that the genes controlling these two traits are distributed across multiple chromosomes, which is a typical feature of polygenic control with incomplete linkage.

In addition, F_2_ segregation showed different association effect sizes between leaf color and chemotype in three independent crosses from different parental individuals. The association effect was strong in Z01 (φ = 0.564) and moderate in Z03 (φ = 0.289), yet nearly absent in Z02 (φ = 0.012). This may result from the homozygosity difference in the parent plant. We provide a foundation for genetic hybridization experiments, which could lead to results deviating from theoretical laws, manifested as significant and irregular differences in the segregation ratio, linkage strength, and recombination rate among different hybrid combinations.

## 4. Conclusions and Outlook for Perilla Breeding and Research

Taken together, the results of this study are of great significance for the breeding of perilla. They overturn the long-standing assumption of a tight linkage between green leaves and the PL type. A new germplasm was created, which combines the high-value PL type with the ideal purple-leaf phenotype. This provides a practical genetic record for the PA × PL combination, the construction of three genetic populations, and valuable experimental material for in-depth research on the molecular mechanisms regulating secondary metabolism, such as the recessive mechanism of PL-type plants and leaf coloration. Moreover, it is important to recognize that such tight correlations between green-leaf and PL-type plants can be influenced by population structure, geography, and human selection, potentially producing an “apparent association” without tight genetic linkage [28].

It is interesting and notable that the positions of the core synthetic gene *GeDH* of the PL pathway and the key transcription factor PfMYB113b [22] for anthocyanin synthesis on the genome are relatively close (Figure 5). It is easy to speculate that genomic structural variations may simultaneously affect the biosynthetic pathways and leaf color chemical types of PL. However, as the last-step catalytic gene of the PL chemotype biosynthetic pathway has not yet been identified, this speculation still needs further verification. Formal genetic mapping [29]/QTL [30] analysis and functional validation of candidate loci are needed in the future. Last but not least, molecular markers need to be developed to accelerate the breeding of new perilla varieties with ideal chemotype and leaf color combinations.

## 5. Materials and Methods

### 5.1. Plant Materials and Crossing Design

Three PA-type *P. frutescens* plants exhibiting purple pigmentation on both the adaxial and abaxial leaf surfaces were used as male parents (IDs: 12, 25, and 14). Three green-leaf PL-type *P. frutescens* plants were used as female parents (IDs: D4, D8, and D7). Controlled crosses were performed in Beijing, China, during September–October 2022 to generate three independent hybrid populations: Z01 (D4, female PL-type × 12, male PA-type), Z02 (D8, female PL-type × 25, male PA-type), and Z03 (D7, female PL-type × 14, male PA-type). Plants 12 and 25 were the cultivated variety from Mishima-shi of Japan, while 14 was the cultivated variety of Hanzhong in Shaanxi, China. D4, D8 and D7 were the cultivated variety from Chongqing of China. Their phenotypes and chemotypes were observed to be stable through two independent growth generations to ensure the reliability of the parental materials for subsequent segregation analysis [31].

As a mainly self-pollinating plant [32], for controlled pollination, the best stages for emasculation and pollination are summarized by a diagram showing the development status of different flowers on *P. frutescens* inflorescences (Supplementary Figure S3). Female flowers were emasculated before anthesis and immediately bagged to prevent pollen contamination. On the following day, pollen from the designated male parent was applied to the emasculated female flowers, and each flower was pollinated once. Hybrid seeds were harvested in late November 2022 and used for subsequent generation advancement and phenotyping/chemotype analyses.

### 5.2. Generation Advancement and Experimental Timeline

The seeds of parental plants were sown in the greenhouse in March and were transplanted to the field in May 2022 with plant spacing of 60 cm for each plant. They were transplanted into the greenhouse on 20 September for hybridization at florescence during September–October 2022. Hybrid seeds were harvested in late November 2022.

The F_1_ generation was sown in the greenhouse in mid-December 2022 in Sanya, Hainan, China. Then they were transplanted to the field in February with plant spacing of 60 cm for each plant. Six F_1_ plants (*n* = 6) were evaluated for leaf color and chemotype by GC-FID (Agilent Technologies Inc., Santa Clara, CA, USA). Self-crossing seeds from confirmed F_1_ individuals were harvested in March 2023 and used to establish the F_2_ generation.

The seeds of F_2_ plants were sown in the greenhouse in April 2023 and were transplanted to the field in May with plant spacing of 60 cm for each plant. The leaf color and chemotype of all F_2_ individuals were identified by GC-FID. Self-crossing seeds from selected F_2_ individuals were collected in November 2023 and used to establish the F_3_ generation, which was grown in Sanya in mid-December 2023 for phenotyping and chemotype analysis with the same procedure as that for the F_1_ generation.

Self-crossing seeds from selected F_3_ individuals were used to establish the F_4_ generation; F_4_ seedlings were raised in Beijing in April 2024, transplanted to the field in May 2024, and evaluated for leaf color and chemotype. In November 2024, self-crossing seeds from selected F_4_ individuals were collected and used to establish the F_5_ generation, which was grown in a greenhouse in Beijing in mid-December 2024 for phenotyping and chemotype analysis.

To support lineage-level tracking of chemotype stability, targeted family sampling was conducted as follows. In F_3_, eight individual F_2_ plants were selected from Z01 to establish the F_3_ population, and six progeny per family were analyzed by GC-FID. In addition, two larger F_3_ populations were established from two PL-type F_2_ individuals within Z01 and analyzed at a higher sampling depth (*n* = 26 and *n* = 45, respectively). In F_4_, six individuals selected from the previously characterized F_3_ population were advanced, and 26 progeny per family were analyzed by GC-FID. In F_5_, 20 F_4_ individuals with known chemotypes were advanced, and one F_5_ progeny plant per F_4_-derived line was randomly selected for chemotype confirmation.

### 5.3. Leaf Color Phenotyping

Leaf color was based on visible leaf color. Scoring was performed using fully expanded leaves evaluated on both adaxial and abaxial surfaces. Leaf color segregation was observed only in F_2_ populations; therefore, analyses of the association between leaf color and chemotype were restricted to the F_2_ generation, where both traits were segregated. For lineage tracking in subsequent generations (F_3_–F_5_), leaf color was recorded for selected individuals alongside chemotype determination.

### 5.4. Leaf Sampling, Extraction, and GC Analysis

#### 5.4.1. Leaf Sampling and N-Hexane Extraction

For chemotype determination, each individual plant was treated as one biological sample. Comparing the relative content in parallel with the same leaf area is only for qualitative detection of the chemotype. A circular piece 9 mm in diameter was punched from the central portion of a fully expanded fresh leaf at the top of the main stem for each sample, avoiding primary and secondary veins. A total of 10 pieces with the same area per plant were immediately soaked in 2 mL of n-hexane (Aladdin Biochemical Technology Co., Ltd., Shanghai, China) in a 5 mL tube and extracted at 4 °C overnight. The extract was filtered through a 0.22 μm membrane (Millipore Sigma, Billerica, MA, USA), transferred to a 2 mL vial, and stored at −20 °C until GC-FID analysis.

#### 5.4.2. GC–FID Analysis

GC-FID analysis was performed on an Agilent 8890 system (Agilent Technologies Inc., Santa Clara, CA, USA) equipped with a flame ionization detector (FID; 300 °C) and an HP-5MS capillary column (30 m × 0.32 mm × 0.25 μm;Agilent Technologies Inc., Santa Clara, CA, USA). Each extract (1 μL) was injected in splitless mode, with the injector temperature set at 200 °C. The oven temperature program was as follows: initial temperature of 50 °C, which was raised to 100 °C at 10 °C min^−1^ and held for 3 min. Then, temperature was raised to 200 °C at 5 °C min^−1^ and held for 3 min. Nitrogen was used as the carrier gas at a flow rate of 1.0 mL min^−1^. Hydrogen (fuel) and air (oxidant) flows for the FID were set according to the manufacturer’s recommendations.

### 5.5. Identification of Chemotype

#### Identification of Perillaldehyde (PA) and Perillene (PL) with Standard Reference Chemical

Perillaldehyde (PA) and perillene (PL) were identified by using peaks corresponding to PA and PL, which were identified by matching retention times with standard reference chemicals analyzed under the same GC-FID conditions. Relative abundances were calculated using only the two marker peaks by area normalization: (1) PA% = PA/(PA + PL) × 100; (2) PL% = PL/(PA + PL) × 100. Values below the quantification limit were recorded as 0. Individuals were classified into four types: (a) Pure PA-type: PA% =100% and PL% = 0; (b) PA-type with trace PL: PA% ≥ 90% and 0 < PL% < 10%; (c) Pure PL-type: PL% = 100% and PA% = 0; and (d) PL-type with trace PA: PL% ≥ 90% and 0 < PA% < 10%. Thus, the individuals were identified as PA-type and PL-type.

### 5.6. Statistical Analysis

The χ^2^ goodness-of-fit test was used to evaluate whether the observed segregation ratios in each F_2_ population conformed to the expected 3:1 ratio under a single-gene dominant inheritance model. Degrees of freedom were 1 for all tests. A heterogeneity χ^2^ test was performed to examine whether the segregation ratios from different crosses could be pooled.

Leaf color and chemotype association analyses were restricted to the F_2_ generation, where both traits were segregated. Associations between leaf color (purple and green) and chemotype (PA and PL) were evaluated using 2 × 2 contingency tables. Pearson’s χ^2^ test was used when all expected counts were ≥5; otherwise, Fisher’s exact test (two-sided) was applied. Association strength was quantified using the φ coefficient (effect size). Analyses were performed separately for each F_2_ population (Z01–Z03) and on the pooled F_2_ dataset. Recombinant frequency was calculated as the proportion of recombinant individuals within each F_2_ population. All statistical analyses were performed in SPSS Statistics v20.0 (IBM, Armonk, NY, USA), with *p* < 0.05 considered statistically significant.

### 5.7. Mapping of Genes Associated with Anthocyanin Biosynthesis (Leaf Color) and Volatile Chemotype

Genes associated with anthocyanin biosynthesis (leaf color) and volatile chemotype were curated from the published literature and GenBank. Gene sequences were aligned to the coding sequences (CDSs) of the *P. frutescens* cv. Hoko-3 reference genome [16]. For each gene model, only the longest CDS was retained. Alignments were performed using TBtools software (v2.363), and only matches with a sequence identity of >95% were accepted. Chromosome identifiers and physical positions (Mb) of retained loci were recorded. A summary of genes is provided in Table 3, and the complete locus list is provided in Supplementary Table S12.

## Figures and Tables

**Table 1:** Segregation of chemotypes in F_2_ progeny of PA-type and PL-type *P. frutescens*.

		F_2_				
Crossing	F_1_ Chemotype	Chemotype	Observed Ratio	Expect Ratio	χ^2^	*p* Value
Z01	PA	PA:PL	83:35	3:1	1.367	0.242
Z02	PA	PA:PL	97:20	3:1	3.9	0.048
Z03	PA	PA:PL	70:18	3:1	0.97	0.325

Note: χ^2^ values were calculated using goodness-of-fit test for each cross independently against the expected 3:1 ratio. *p* values were derived from the χ^2^ distribution with 1 degree of freedom. A heterogeneity χ^2^ test across the three crosses gave χ^2^ = 5.245 (df = 2, *p* > 0.05), indicating no significant difference among three crosses, allowing the data to be pooled.

**Table 2:** Leaf color × chemotype contingency tables in F_2_ populations of *P. frutescens*.

Population	*n*	Green-Leaf PA-Type	Green-Leaf PL-Type	Purple-Leaf PA-Type	Purple-Leaf PL- Type	χ^2^	*p*	φ	Recombinants ^1^	Recombinant Rate
Z01	118	9	23	74	12	37.5	9.13 × 10^−10^	0.564	21	17.80%
Z02	117	50	10	47	10	0.016	0.9	0.012	60	51.30%
Z03	88	16	10	54	8	7.35	0.00669	0.289	24	27.30%
Pooled	323	75	43	175	30	20.359	6.42 × 10^−6^	0.251	105	32.50%

^1^ green-leaf PA-type and purple-leaf PL-type *P. frutescens.*

**Table 3:** Homologous genes of leaf color and chemotypes in *P. frutescens*.

Trait	Gene	Gene Family	GenBank Accession No.	Reference
Leaf color	*Pf3-GT*	Flavonoid 3-O-glucosyltransferase	AB002818.1	[18]
Leaf color	*Pf5-GT1*	Anthocyanin 5-O-glucosyltransferase	AB013596.1	[19]
Leaf color	*Pf5-GT2*	Anthocyanin 5-O-glucosyltransferase	AB013598.1	[19]
Leaf color	*PfACT*	Anthocyanin acyltransferase	AB029340.1	[20]
Leaf color	*PfMAT*	Anthocyanin malonyltransferase	AF489108.1	[21]
Leaf color	*PfANS*	Anthocyanidin synthase	AB003779.1	[20]
Leaf color	*PfCHI*	Chalcone isomerase	AB362192.1	[21]
Leaf color	*PfC4H1*	Cinnamate-4-hydroxylase	-	[22]
Leaf color	*PfCHS1*	Chalcone synthase	AB002582.1	[18]
Leaf color	*PfCHS2*	Chalcone synthase	AB002815.1	[18]
Leaf color	*PfDFR*	Dihydroflavonol reductase	AB002817.1	[18]
Leaf color	*PfF3′H*	Flavonoid 3′-hydroxylase	AB045593.1	[20]
Leaf color	*PfF3H1*	Flavanone 3-hydroxylase	AB002816.1	[18]
Leaf color	*PfF3H*	Flavonoid-3-hydroxylase	-	[22]
Leaf color	*PfMYB113b*	chr08_11487857_11491178	-	[22]
Chemotype	*PfAKR*	Aldo-keto reductase	JX629451	[23]
Chemotype	*PcAKR*	Aldo-keto reductase	JX629452	[23]
Chemotype	*PsAKR*	Aldo-keto reductase	JX629453	[23]
Chemotype	*PfGeDH*	Geraniol dehydrogenase	JX855836	[23]
Chemotype	*PcGeDH*	Geraniol dehydrogenase	JX855837	[23]
Chemotype	*PsGeDH*	Geraniol dehydrogenase	JX855838	[23]
Chemotype	*PcPT-ISPD2*	Isopiperitenol dehydrogenase	KF766528	[23]
Chemotype	*LC*	Limonene cyclase	D49368	[13]
Chemotype	*PcTps-C-GS*	Geraniol synthase	DQ088667	[10]
Chemotype	*PfTps-PL-GS*	Geraniol synthase	DQ234300	[10]
Chemotype	*PcTps-EK-GS*	Geraniol synthase	DQ234299	[10]
Chemotype	*PcTps-PK-GS*	Geraniol synthase	DQ234298	[10]
Chemotype	*PhTps-5073G-GS*	Geraniol synthase	FJ644547	[24]
Chemotype	*PsTps-5031G-GS*	Geraniol synthase	FJ644545	[24]
Chemotype	*PTS-5526-MS*	Myrcene synthase	AF271259	[25]
Chemotype	*CYP71D174*	(−)-limonene-7-hydroxylase	GQ120438	[14]
Chemotype	*CYP71AT146*	Perillalcohol/perillaldehyde synthase	KU674339	[15]

## Data Availability

The original contributions presented in this study are included in the article/supplementary material. Further inquiries can be directed to the corresponding authors.

## Supplementary Materials

The following supporting information can be downloaded at: https://www.mdpi.com/article/10.3390/plants15071044/s1, Figure S1. The chromatogram of standard substance of perillaldehyde; Figure S2. The chromatogram of standard substance of perillene; Figure S3. A diagram showing the development status of different flowers on *P. frutescens* inflorescences. The flower of stage 4 is suitable for hybridization; Table S1. Standard substance of perillaldehyde identified by GC-FID; Table S2. *P. frutescens* plants with purple leaves numbered 12, 25 and 14 (Figure 1) were identified by GC-FID as PA-type; Table S3. Standard substance of perillene identified by GC-FID; Table S4. *P. frutescens* plants with green leaves numbered D4, D8 and D7 (Figure 1) were identified by GC-FID as PL-type; Table S5. GC-FID analysis showed that chemotypes of F_1_ individuals in three crosses were all PA-type plants; Table S6. Chemotype and leaf color identification of individuals in F_2_ populations of Z01; Table S7. Chemotype and leaf color identification of individuals in F_2_ populations in Z02; Table S8. Chemotype and leaf color identification of individuals in F_2_ populations of Z03; Table S9. Chemotype and leaf color identification of individuals in F_3_ populations of Z01; Table S10. Chemotype and leaf color identification of individuals in F_4_ populations of Z01; Table S11. Chemotype and leaf color identification of individuals in F_5_ populations of Z01; Table S12. Chromosomal distribution information of genes associated with leaf color and chemotypes.
