# Supplementary material for: Development of a Purple-Leaf Perillene Chemotype Line in Perilla frutescens Reveals Incomplete Linkage with Leaf Color
Source: Plants (Basel). 2026 Mar 28;15(7):1044. doi: 10.3390/plants15071044 (PMC13075182; doi:10.3390/plants15071044)
Supplement: Supplementary file 1 [file plants-15-01044-s001.zip › plants-4206551-supplementary.pdf]

Supplementary Material

Figures and Tables

Figure S1. The chromatogram of standard substance of perillaldehyde.

Figure S2. The chromatogram of standard substance of perillene.

Figure S3. A diagram showing the development status of different flowers on *P. frutescens* inflorescences. The flower of stage 4 is suitable for hybridization.

Table S1. Standard substance of perillaldehyde identified by GC-FID.

Table S2. *P. frutescens* plants with purple-leaf numbered 12, 25 and 14 (Figure1) were identified by GC-FID as PA-type.

Table S3. Standard substance of perillene identified by GC-FID.

Table S4. *P. frutescens* plants with green-leaf numbered D4, D8 and D7 (Figure1) were identified by GC-FID as PL-type.

Table S5. GC-FID analysis showed that chemotype of F_1 individuals in three crosses were all PA-type plants.

Table S6. Chemotype and leaf color identification of individuals in F_2 populations of Z01.
Table S7. Chemotype and leaf color identification of individuals in F_2 populations in Z02.
Table S8. Chemotype and leaf color identification of individuals in F_2 populations of Z03.
Table S9. Chemotype and leaf color identification of individuals in F_3 populations of Z01.
Table S10. Chemotype and leaf color identification of individuals in F_4 populations of Z01.

Table S11. Chemotype and leaf color identification of individuals in F_5 populations of Z01.
Table S12. Chromosomal distribution information of genes of leaf color and chemotypes.

Figure S1. The chromatogram of standard substance of perillaldehyde.

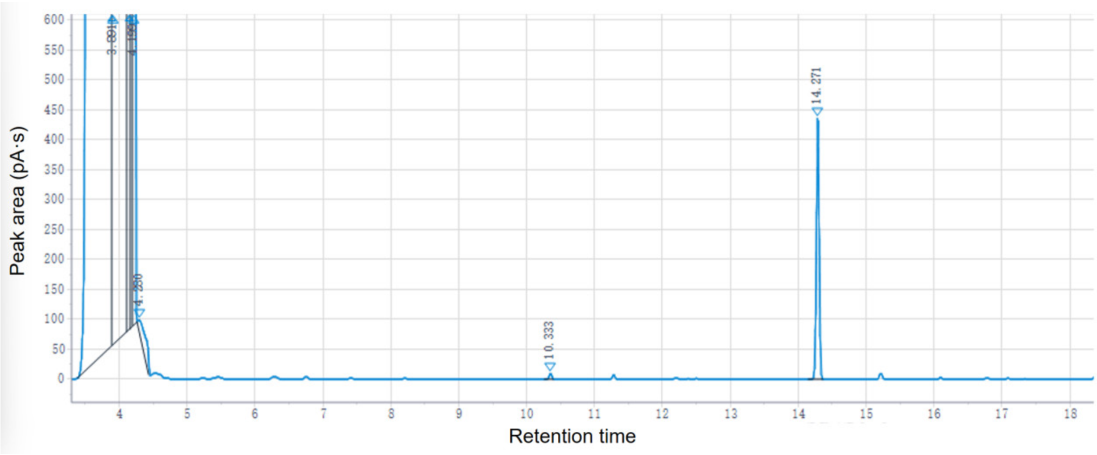

Figure S2. The chromatogram of standard substance of perillene.

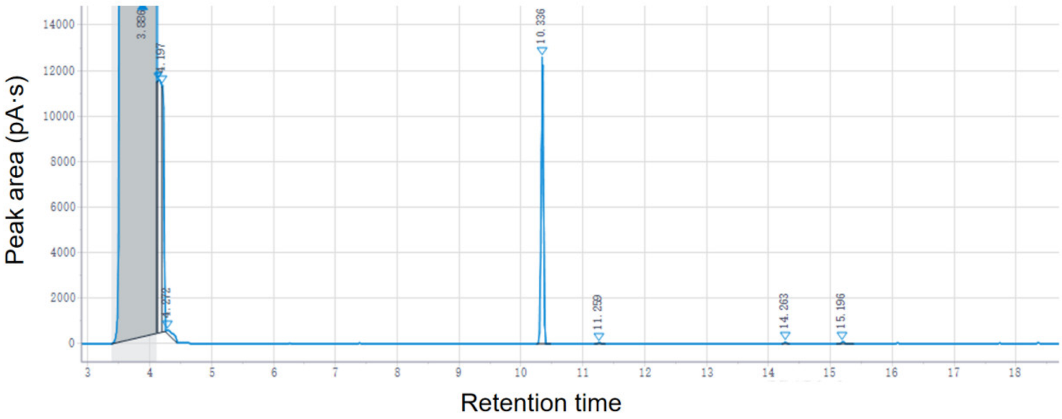

Figure S3. A diagram showing the development status of different flowers on *P. frutescens* inflorescences. The flower of stage 4 is suitable for hybridization.

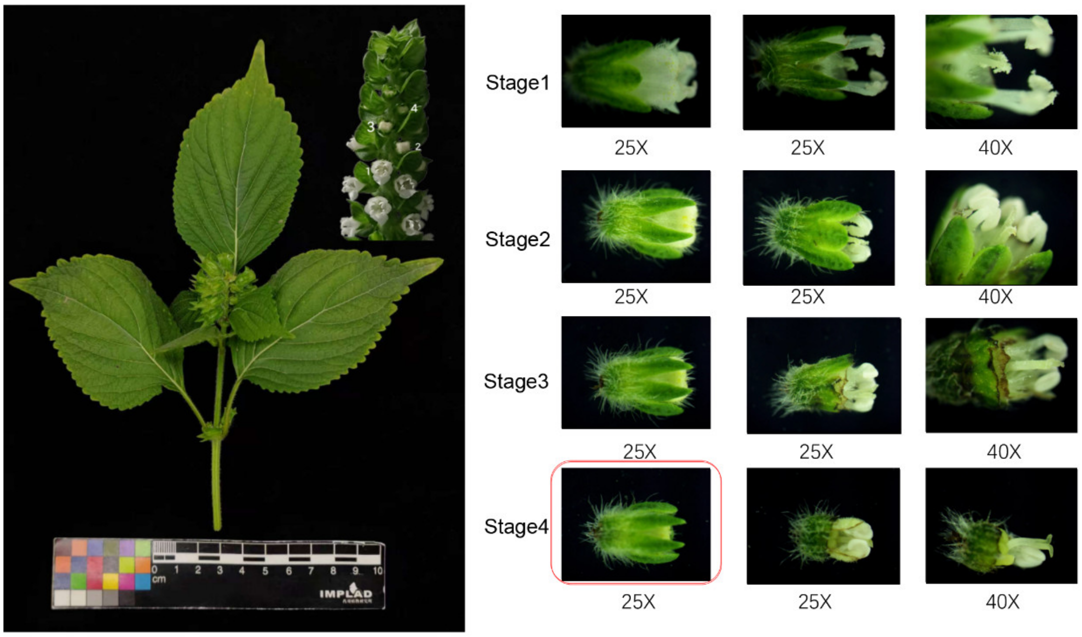

Table S1. Standard substance of perillaldehyde identified by GC-FID.

Name	Signal	RT (min)	Peak area (pA·s)	Peak area %	Peak height (pA)	Start time (min)	End time (min)
-	FID1B	3.881	4203619.389	59.339	340481.955	3.377	3.886
-	FID1B	3.891	2878068.734	40.627	340483.129	3.886	4.101
-	FID1B	4.132	825.279	0.012	305.249	4.101	4.147
-	FID1B	4.158	818.235	0.012	304.31	4.147	4.192
-	FID1B	4.199	484.857	0.007	297.545	4.192	4.245
-	FID1B	4.28	45.754	0.001	3.369	4.245	4.43
-	FID1B	10.333	5.081	0	1.871	10.247	10.394
Perillaldehyde	FID1B	14.271	191.967	0.003	70.791	14.137	14.36

Table S2. *P. frutescens* plants with purple-leaf numbered 12, 25 and 14 (Figure1) were identified by GC-FID as PA-type.

Sample	Retention time	Peak area
PA standard	14.279	191.967
12	14.279	419.322
25	14.279	465.563
14	14.279	148.678

Table S3. Standard substance of perillene identified by GC-FID.

Name	Signal	RT (min)	Peak area (pA·s)	Peak area %	Peak height (pA)	Peak height %	Start time (min)	End time (min)
Perillene	FID1B	3.886	7215801.381	99.957	348293.104	99.64	3.377	4.103
	FID1B	4.125	482.771	0.007	306.631	0.09	4.103	4.129
	FID1B	4.141	1057.214	0.015	307.201	0.09	4.129	4.187
	FID1B	4.197	582.017	0.008	299.286	0.09	4.187	4.246
	FID1B	4.272	45.338	0.001	2.81	0	4.246	4.43
	FID1B	10.336	886.814	0.012	348.688	0.1	10.255	10.483
	FID1B	11.259	5.568	0	2.079	0	11.19	11.36
	FID1B	14.263	5.867	0	2.134	0	14.213	14.333
	FID1B	15.196	8.939	0	2.68	0	15.103	15.377
	FID1B	19.208	8.318	0	2.085	0	19	19.294

Table S4. *P. frutescens* plants with green-leaf numbered D4, D8 and D7 (Figure1) were identified by GC-FID as PL-type.

Sample	Retention time	Peak area
Perillene standard substance	10.355	886.814
D4	10.355	170.698
D8	10.355	111.483
D7	10.355	150.973

Table S5. GC-FID analysis showed that chemotype of F_1 individuals in three crosses were all PA-type plants.

Sample Name	Perillene	Perillaldehyde	Chemotype	Leaf color
Z01F_1(1)	4.345	264.594	PA	Mixed purple
Z01F_1(2)	0	22.165	PA	Mixed purple
Z02F_1(1)	0	627.86	PA	Mixed purple
Z02F_1(2)	19.94	178.417	PA	Mixed purple
Z03F_1(1)	0	158.091	PA	Mixed purple
Z03F_1(2)	7.642	285.362	PA	Mixed purple

Table S6. Chemotype and leaf color identification of individuals in F_2 populations of Z01.

Sample Name	Perillene	Perillaldehyde	Chemotype	Leaf color
Z01-2	247.048	0	PL	Purple
Z01-3	280.715	0	PL	Purple
Z01-4	249.716	0	PL	Purple
Z01-6	247.444	0	PL	Green
Z01-7	233.629	0	PL	Green
Z01-9	1380.311	28.048	PL	Purple
Z01-10	369.281	7.9	PL	Green
Z01-15	768.189	13.535	PL	Green
Z01-16	439.423	7.636	PL	Green
Z01-22	726.121	10.058	PL	Green
Z01-24	613.964	9.787	PL	Green
Z01-27	796.277	10.341	PL	Green
Z01-28	646.469	66.717	PL	Green
Z01-31	611.427	17.609	PL	Green
Z01-32	447.222	6.885	PL	Green
Z01-45	540.223	11.652	PL	Green
Z01-48	533.051	17.717	PL	Green
Z01-49	617.917	11.1	PL	Purple
Z01-52	572.358	15.91	PL	Green
Z01-56	488.351	18.661	PL	Green
Z01-58	617.3	23.949	PL	Green
Z01-62	601.812	0	PL	Green
Z01-64	310.673	0	PL	Purple
Z01-67	576.033	0	PL	Green
Z01-75	522.804	0	PL	Purple
Z01-78	469.425	0	PL	Purple
Z01-83	655.081	0	PL	Green
Z01-84	494.387	0	PL	Green
Z01-85	626.157	0	PL	Purple
Z01-91	515.974	0	PL	Green
Z01-94	568.588	0	PL	Purple
Z01-95	494.729	4.817	PL	Green
Z01-96	271.447	7.889	PL	Green
Z01-107	615.863	0	PL	Purple
Z01-116	540.605	0	PL	Purple
Z01-1	6.718	308.111	PA	Purple
Z01-5	4.972	454.044	PA	Purple
Z01-8	0	365.169	PA	Purple
Z01-11	11.576	469.47	PA	Purple
Z01-12	16.704	473.039	PA	Purple
Z01-13	11.48	323.301	PA	Purple

Z01-14	5.207	332.367	PA	Purple
Z01-17	8.859	252.377	PA	Purple
Z01-18	8.739	462.955	PA	Purple
Z01-19	8.278	335.812	PA	Purple
Z01-20	14.827	306.673	PA	Purple
Z01-21	8.113	679.96	PA	Purple
Z01-23	23.188	1044.662	PA	Purple
Z01-25	17.2	350.907	PA	Purple
Z01-26	15.441	696.038	PA	Purple
Z01-29	11.046	323.192	PA	Purple
Z01-30	12.463	770.94	PA	Purple
Z01-33	33.047	509.537	PA	Purple
Z01-34	30.584	551.921	PA	Purple
Z01-35	11.712	443.459	PA	Purple
Z01-36	19.292	482.427	PA	Purple
Z01-37	14.404	524.508	PA	Purple
Z01-38	11.312	333.233	PA	Purple
Z01-39	0	242.086	PA	Purple
Z01-40	22.516	693.559	PA	Purple
Z01-41	7.746	1110.161	PA	Purple
Z01-42	17.655	370.476	PA	Purple
Z01-43	10.941	337.311	PA	Purple
Z01-44	10.379	333.081	PA	Purple
Z01-46	24.565	464.512	PA	Purple
Z01-47	26.555	610.385	PA	Purple
Z01-50	25.561	774.224	PA	Purple
Z01-51	15.1	506.16	PA	Purple
Z01-53	0	488.498	PA	Purple
Z01-54	0	545.614	PA	Purple
Z01-55	9.76	849.737	PA	Purple
Z01-57	17.19	1157.256	PA	Purple
Z01-59	33.988	792.304	PA	Purple
Z01-60	4.339	1116.354	PA	Purple
Z01-61	0	196.574	PA	Purple
Z01-63	0	435.172	PA	Purple
Z01-65	8.531	270.492	PA	Purple
Z01-66	0	220.066	PA	Purple
Z01-68	0	182.133	PA	Purple
Z01-69	0	221.975	PA	Purple
Z01-70	6.938	145.699	PA	Purple
Z01-71	8.143	320.274	PA	Purple
Z01-72	0	368.173	PA	Purple
Z01-73	0	355.567	PA	Purple
Z01-74	6.962	225.695	PA	Green

Z01-76	0	256.714	PA	Purple
Z01-77	6.373	281.418	PA	Purple
Z01-79	0	208.81	PA	Purple
Z01-80	0	218.964	PA	Purple
Z01-81	5.991	600.363	PA	Purple
Z01-82	0	235.795	PA	Purple
Z01-86	0	122.617	PA	Purple
Z01-87	0	207.591	PA	Purple
Z01-88	0	246.861	PA	Purple
Z01-89	0	533.169	PA	Purple
Z01-90	0	312.214	PA	Purple
Z01-92	6.833	298.481	PA	Purple
Z01-93	0	286.461	PA	Green
Z01-97	0	137.253	PA	Purple
Z01-98	0	571.686	PA	Purple
Z01-99	0	463.021	PA	Purple
Z01-100	0	425.223	PA	Green
Z01-101	0	269.093	PA	Purple
Z01-102	0	513.463	PA	Purple
Z01-103	6.916	210.884	PA	Purple
Z01-104	5.634	953.707	PA	Purple
Z01-105	5.32	320.076	PA	Purple
Z01-106	0	310.342	PA	Purple
Z01-108	6.799	510.457	PA	Purple
Z01-109	0	173.833	PA	Purple
Z01-110	5.68	349.61	PA	Purple
Z01-111	6.467	347.724	PA	Green
Z01-112	0	606.095	PA	Green
Z01-113	5.308	280.379	PA	Green
Z01-114	8.368	313.937	PA	Purple
Z01-115	6.2	202.317	PA	Green
Z01-117	12.468	304.124	PA	Green
Z01-118	34.345	468.433	PA	Green

Table S7. Chemotype and leaf color identification of individuals in F_2 populations in Z02.

Sample Name	Perillene	Perillaldehyde	Chemotype	Leaf color
Z02-15	484.196	13.653	PL	Purple
Z02-17	276.872	6.936	PL	Purple
Z02-18	323.851	11.659	PL	Purple
Z02-30	406.422	12.881	PL	Purple
Z02-36	362.431	14.512	PL	Purple
Z02-39	630.801	5.763	PL	Purple
Z02-49	825.067	11.245	PL	Purple
Z02-56	485.398	12.837	PL	Purple
Z02-69	440.299	6.726	PL	Green
Z02-71	525.94	0	PL	Purple
Z02-77	1020.197	14.023	PL	Green
Z02-79	603.553	10.463	PL	Green
Z02-84	759.441	8.166	PL	Green
Z02-85	405.562	7.368	PL	Green
Z02-92	539.16	0	PL	Green
Z02-96	387.421	0	PL	Green
Z02-99	394.162	6.257	PL	Green
Z02-108	865.658	0	PL	Green
Z02-110	343.403	0	PL	Green
Z02-121	0	49.118	PL	Purple
Z02-2	6.302	190.013	PA	Purple
Z02-5	0	237.955	PA	Purple
Z02-6	11.151	335.689	PA	Purple
Z02-7	7.887	299.712	PA	Purple
Z02-8	8.887	654.858	PA	Purple
Z02-9	8.228	232.017	PA	Purple
Z02-10	0	295.369	PA	Purple
Z02-11	0	429.483	PA	Purple
Z02-12	7.755	607.523	PA	Purple
Z02-13	0	266.793	PA	Purple
Z02-14	0	269.469	PA	Purple
Z02-16	0	339.54	PA	Purple
Z02-19	8.306	301.511	PA	Purple
Z02-20	9.137	354.088	PA	Purple
Z02-21	9.446	300.072	PA	Purple
Z02-22	0	354.595	PA	Purple
Z02-23	0	613.909	PA	Purple
Z02-24	8.184	1030.666	PA	Green
Z02-26	0	524.722	PA	Purple
Z02-27	5.766	385.292	PA	Purple
Z02-28	0	873.61	PA	Purple

Z02-29	0	803.563	PA	Purple
Z02-31	8.643	398.796	PA	Purple
Z02-32	4.183	651.116	PA	Purple
Z02-33	0	227.857	PA	Purple
Z02-34	9.248	319.146	PA	Purple
Z02-35	0	393.042	PA	Purple
Z02-37	0	729.406	PA	Purple
Z02-42	0	535.413	PA	Purple
Z02-43	0	653.956	PA	Purple
Z02-44	0	490.601	PA	Purple
Z02-45	0	377.557	PA	Purple
Z02-46	12.375	529.432	PA	Purple
Z02-48	9.57	878.47	PA	Purple
Z02-50	8.867	1576.963	PA	Green
Z02-51	0	642.212	PA	Purple
Z02-52	9.18	159.79	PA	Purple
Z02-53	0	351.999	PA	Purple
Z02-54	0	463.049	PA	Purple
Z02-55	14.202	373.679	PA	Purple
Z02-57	8.151	417.561	PA	Green
Z02-58	6.181	340.234	PA	Green
Z02-59	0	1149.335	PA	Purple
Z02-60	0	829.375	PA	Purple
Z02-61	0	416.866	PA	Green
Z02-62	0	314.045	PA	Purple
Z02-63	0	257.499	PA	Purple
Z02-64	0	432.667	PA	Green
Z02-65	0	1099.945	PA	Green
Z02-66	14.792	520.593	PA	Purple
Z02-67	0	493.329	PA	Green
Z02-68	0	508.45	PA	Green
Z02-70	0	376.058	PA	Green
Z02-72	0	254.283	PA	Green
Z02-73	0	392.789	PA	Purple
Z02-74	0	498.414	PA	Green
Z02-75	0	186.696	PA	Green
Z02-76	0	624.48	PA	Green
Z02-78	39.093	1075.213	PA	Green
Z02-80	0	604.843	PA	Purple
Z02-81	0	1150.827	PA	Green
Z02-82	20.361	1153.828	PA	Green
Z02-83	28.943	696.943	PA	Green
Z02-86	0	144.384	PA	Green
Z02-88	0	235.307	PA	Green

Z02-89	0	237.913	PA	Green
Z02-90	8.884	381.693	PA	Green
Z02-91	0	280.19	PA	Green
Z02-93	8.487	434.044	PA	Green
Z02-94	8.181	405.177	PA	Green
Z02-95	5.985	312.56	PA	Green
Z02-97	0	346.35	PA	Green
Z02-98	0	310.79	PA	Green
Z02-100	0	1136.701	PA	Green
Z02-101	0	373.466	PA	Green
Z02-102	0	815.107	PA	Green
Z02-103	0	240.791	PA	Green
Z02-104	17.411	456.802	PA	Green
Z02-105	0	277.713	PA	Green
Z02-106	11.233	324.801	PA	Green
Z02-107	0	244.603	PA	Green
Z02-109	0	439.726	PA	Green
Z02-111	0	393.478	PA	Green
Z02-112	0	245.676	PA	Green
Z02-113	0	430.739	PA	Purple
Z02-114	0	450.223	PA	Green
Z02-115	0	326.645	PA	Green
Z02-116	0	517.588	PA	Green
Z02-117	0	551.328	PA	Green
Z02-118	0	309.992	PA	Green
Z02-119	0	658.386	PA	Green
Z02-120	0	246.867	PA	Purple
Z02-122	0	769.169	PA	Green
Z02-123	0	211.156	PA	Green
Z02-124	0	688.59	PA	Green
Z02-125	0	637.593	PA	Green
Z02-126	0	1154.645	PA	Green

Table S8. Chemotype and leaf color identification of individuals in F_2 populations of Z03.

Sample Name	Perillene	Perillaldehyde	Chemotype	Leaf color
Z03-1	27.284	780.872	PA	Purple
Z03-2	0	422.644	PA	Purple
Z03-3	0	464.107	PA	Purple
Z03-5	9.455	603.021	PA	Purple
Z03-6	15.914	436.317	PA	Purple
Z03-11	10.971	228.806	PA	Purple
Z03-12	11.204	422.308	PA	Purple
Z03-16	0	289.803	PA	Purple
Z03-18	7.098	430.911	PA	Purple
Z03-19	0	314.327	PA	Purple
Z03-20	0	476.473	PA	Purple
Z03-21	0	695.035	PA	Purple
Z03-22	13.401	344.07	PA	Purple
Z03-23	24.182	472.987	PA	Purple
Z03-24	0	672.069	PA	Purple
Z03-25	0	488.949	PA	Purple
Z03-26	10.448	272.47	PA	Purple
Z03-27	4.073	490.358	PA	Purple
Z03-29	0	178.319	PA	Purple
Z03-30	0	366.021	PA	Purple
Z03-32	0	268.476	PA	Purple
Z03-33	0	395.124	PA	Purple
Z03-34	0	429.538	PA	Purple
Z03-35	0	437.29	PA	Purple
Z03-36	16.193	444.262	PA	Purple
Z03-39	0	194.232	PA	Purple
Z03-40	0	367.033	PA	Purple
Z03-43	7.784	342.907	PA	Purple
Z03-45	0	238.464	PA	Purple
Z03-46	11.883	599.003	PA	Purple
Z03-47	7.101	937.394	PA	Purple
Z03-48	0	690.636	PA	Purple
Z03-49	8.641	368.937	PA	Purple
Z03-50	0	328.145	PA	Purple
Z03-51	5.606	495.783	PA	Purple
Z03-53	0	491.684	PA	Purple
Z03-56	0	160.139	PA	Purple
Z03-57	0	131.874	PA	Purple
Z03-58	0	448.298	PA	Purple
Z03-59	0	434.233	PA	Purple
Z03-60	0	460.92	PA	Purple

Z03-61	0	209.846	PA	Green
Z03-62	0	297.933	PA	Green
Z03-63	13.067	407.877	PA	Purple
Z03-65	0	363.364	PA	Purple
Z03-66	0	473.383	PA	Purple
Z03-67	0	257.675	PA	Purple
Z03-68	0	352.241	PA	Purple
Z03-69	4.612	258.295	PA	Purple
Z03-71	0	665.109	PA	Purple
Z03-72	0	237.766	PA	Purple
Z03-73	0	248.947	PA	Purple
Z03-75	0	411.548	PA	Purple
Z03-76	0	410.289	PA	Purple
Z03-77	0	614.786	PA	Green
Z03-78	0	686.345	PA	Green
Z03-79	0	345.797	PA	Green
Z03-81	11.87	478.71	PA	Green
Z03-83	0	196.41	PA	Green
Z03-85	11.461	429.034	PA	Green
Z03-86	7.193	331.121	PA	Green
Z03-89	7.967	691.784	PA	Green
Z03-90	5.825	342.378	PA	Green
Z03-91	6.494	410.993	PA	Purple
Z03-94	0	749.363	PA	Green
Z03-96	0	239.324	PA	Green
Z03-97	0	168.446	PA	Green
Z03-98	0	275.572	PA	Green
Z03-99	6.699	389.258	PA	Green
Z03-17	19.866	465.043	PA	Purple
Z03-4	340.59	5.236	PL	Purple
Z03-8	732.535	0	PL	Purple
Z03-28	354.863	0	PL	Purple
Z03-37	860.033	0	PL	Purple
Z03-41	295.078	0	PL	Purple
Z03-42	206.186	0	PL	Purple
Z03-52	468.809	16.357	PL	Green
Z03-70	532.124	0	PL	Purple
Z03-74	354.231	0	PL	Purple
Z03-80	473.235	7.869	PL	Green
Z03-82	309.492	9.484	PL	Green
Z03-87	276.753	0	PL	Green
Z03-88	252.816	0	PL	Green
Z03-92	310.538	0	PL	Green
Z03-93	399.955	0	PL	Green

Z03-95	276.023	0	PL	Green
Z03-100	275.939	0	PL	Green

Table S9. Chemotype and leaf color identification of individuals in F_3 populations of Z01.

Sample Name	Perillene	Perillaldehyde	Chemotype	Leaf color
Z01-1-1	0	695.168	PA	Purple
Z01-1-2	0	1702.333	PA	Purple
Z01-1-3	0	858.263	PA	Purple
Z01-1-4	0	960.043	PA	Purple
Z01-1-5	0	1089.76	PA	Purple
Z01-1-6	0	1054.51	PA	Purple
Z01-2-1	655.835	0	PL	Purple
Z01-2-2	687.19	0	PL	Purple
Z01-2-3	362.381	0	PL	Purple
Z01-2-4	585.545	0	PL	Purple
Z01-2-5	638.708	0	PL	Purple
Z01-2-6	266.471	0	PL	Purple
Z01-4-1	141.102	0	PL	Purple
Z01-4-2	209.167	0	PL	Purple
Z01-4-3	136.112	0	PL	Purple
Z01-4-4	223.914	0	PL	Purple
Z01-4-5	185.57	0	PL	Purple
Z01-4-6	142.563	0	PL	Purple
Z01-9-1	387.046	0	PL	Purple
Z01-9-2	374.325	0	PL	Purple
Z01-9-3	386.048	0	PL	Purple
Z01-9-4	343.416	0	PL	Purple
Z01-9-5	467.005	0	PL	Purple
Z01-9-6	565.249	0	PL	Purple
Z01-11-1	0	556.298	PA	Purple
Z01-11-2	0	569.315	PA	Purple
Z01-11-3	0	847.71	PA	Purple
Z01-11-4	0	892.976	PA	Purple
Z01-11-5	0	1152.204	PA	Purple
Z01-11-6	0	153.483	PA	Purple
Z01-15-1	339.682	0	PL	Purple
Z01-15-2	191.214	0	PL	Purple
Z01-15-3	288.377	0	PL	Purple
Z01-15-4	364.396	0	PL	Purple
Z01-15-5	266.061	0	PL	Purple
Z01-15-6	212.493	0	PL	Purple
Z01-91-1	426.112	0	PL	Green
Z01-91-2	320.771	0	PL	Green
Z01-91-3	374.515	0	PL	Green
Z01-91-4	521.623	0	PL	Green
Z01-91-5	455.357	0	PL	Green

Z01-91-6	257.163	0	PL	Green
Z01-95-1	404.947	0	PL	Green
Z01-95-2	344.113	0	PL	Green
Z01-95-3	233.241	0	PL	Green
Z01-95-4	148.456	0	PL	Green
Z01-95-5	451.373	0	PL	Green
Z01-95-6	198.045	0	PL	Green
Z01-95-1	1357.064	0	PL	Green
Z01-95-2	2881.706	0	PL	Green
Z01-95-3	2643.612	0	PL	Green
Z01-95-4	1598.538	0	PL	Green
Z01-95-5	5847.209	0	PL	Green
Z01-95-6	3020.99	0	PL	Green
Z01-95-7	0	1529.524	PA	Green
Z01-95-8	2679.156	0	PL	Green
Z01-95-9	1968.609	0	PL	Green
Z01-95-10	2751.958	0	PL	Green
Z01-95-11	2279.98	0	PL	Green
Z01-95-12	2731.718	0	PL	Green
Z01-95-13	2137.82	0	PL	Green
Z01-95-14	3031.932	0	PL	Green
Z01-95-15	2300.53	0	PL	Green
Z01-95-16	6390.52	0	PL	Green
Z01-95-17	2179.26	0	PL	Green
Z01-95-18	2670.455	0	PL	Green
Z01-95-19	2746.322	0	PL	Green
Z01-95-20	3783.744	0	PL	Green
Z01-95-21	3078.35	0	PL	Green
Z01-95-22	2444.042	0	PL	Green
Z01-95-23	5208.712	0	PL	Green
Z01-95-24	1793.754	0	PL	Green
Z01-95-25	3323.659	0	PL	Green
Z01-95-26	2095.092	0	PL	Green
Z01-15-1	5396.626	0	PL	Green
Z01-15-2	5173.144	0	PL	Green
Z01-15-3	2365.296	0	PL	Green
Z01-15-4	8780.345	0	PL	Green
Z01-15-5	6625.104	0	PL	Green
Z01-15-6	4993.542	0	PL	Green
Z01-15-7	11785.808	0	PL	Green
Z01-15-8	4923.509	0	PL	Green
Z01-15-9	3036.389	0	PL	Green
Z01-15-26	2213.627	0	PL	Green
Z01-15-27	3259.779	0	PL	Green

Z01-15-28	12024.98	0	PL	Green
Z01-15-29	4223.676	0	PL	Green
Z01-15-30	6570.09	0	PL	Green
Z01-15-31	2592.114	0	PL	Green
Z01-15-32	4020.313	0	PL	Green
Z01-15-33	7125.788	0	PL	Green
Z01-15-34	5567.424	0	PL	Green
Z01-15-35	2199.246	0	PL	Green
Z01-15-36	3634.658	0	PL	Green
Z01-15-37	7647.966	0	PL	Green
Z01-15-38	4235.06	0	PL	Green
Z01-15-39	1749.333	0	PL	Green
Z01-15-40	4861.517	0	PL	Green
Z01-15-41	6384.386	0	PL	Green
Z01-15-42	2903.906	0	PL	Green
Z01-15-43	3434.487	0	PL	Green
Z01-15-44	0	2635.526	PA	Green
Z01-15-45	3354.486	0	PL	Green
Z01-15-46	4073.811	0	PL	Green
Z01-15-47	4884.047	0	PL	Green
Z01-15-48	3882.274	0	PL	Green
Z01-15-49	3343.71	0	PL	Green
Z01-15-50	3703.128	0	PL	Green
Z01-15-51	3965.422	0	PL	Green
Z01-15-52	6446.51	0	PL	Green
Z01-15-53	2739.655	0	PL	Green
Z01-15-54	2499.543	0	PL	Green
Z01-15-55	6665.34	0	PL	Green
Z01-15-56	4690.259	0	PL	Green
Z01-15-57	6083.886	0	PL	Green
Z01-15-58	4547.598	0	PL	Green
Z01-15-59	4066.505	0	PL	Green
Z01-15-60	9559.632	0	PL	Green
Z01-15-61	0	2273.789	PA	Green

Table S10. Chemotype and leaf color identification of individuals in F_4 populations of Z01.

Sample Name	Perillene	Perillaldehyde	Chemotype	Leaf color
Z01-2-2-1	3373.662	0	PL	Purple
Z01-2-2-2	3570.522	0	PL	Purple
Z01-2-2-3	3373.662	0	PL	Purple
Z01-2-2-4	11599.76	0	PL	Purple
Z01-2-2-5	8904.582	0	PL	Purple
Z01-2-2-7	1111.313	0	PL	Purple
Z01-2-2-8	1096.639	0	PL	Purple
Z01-2-2-9	969.075	0	PL	Purple
Z01-2-2-10	631.802	0	PL	Purple
Z01-2-2-11	493.266	0	PL	Purple
Z01-2-2-12	3373.662	0	PL	Purple
Z01-2-2-13	3570.522	0	PL	Purple
Z01-2-2-14	3570.522	0	PL	Purple
Z01-2-2-15	3373.662	0	PL	Purple
Z01-2-2-16	3373.662	0	PL	Purple
Z01-2-2-17	2324.162	0	PL	Purple
Z01-2-2-18	3570.522	0	PL	Purple
Z01-2-2-19	2324.162	0	PL	Purple
Z01-2-2-20	3373.662	0	PL	Purple
Z01-2-2-21	2324.162	0	PL	Purple
Z01-2-2-22	2324.162	0	PL	Purple
Z01-2-2-23	2324.162	0	PL	Purple
Z01-2-2-24	2324.162	0	PL	Purple
Z01-2-2-25	3570.522	0	PL	Purple
Z01-2-2-26	3570.522	0	PL	Purple
Z01-2-3-1	7918.319	0	PL	Purple
Z01-2-3-2	17788.877	0	PL	Purple
Z01-2-3-3	14788.245	0	PL	Purple
Z01-2-3-4	16420.8	0	PL	Purple
Z01-2-3-5	17334.777	0	PL	Purple
Z01-2-3-6	1537.181	0	PL	Purple
Z01-2-3-7	590.183	0	PL	Purple
Z01-2-3-8	1633.205	0	PL	Purple
Z01-2-3-9	3106.267	0	PL	Purple
Z01-2-3-10	3106.267	0	PL	Purple
Z01-2-3-11	3084.971	0	PL	Purple
Z01-2-3-12	3084.971	0	PL	Purple
Z01-2-3-13	3084.971	0	PL	Purple
Z01-2-3-14	3084.971	0	PL	Purple
Z01-2-3-15	3084.971	0	PL	Purple
Z01-2-3-16	3084.971	0	PL	Purple

Z01-2-3-17	3106.267	0	PL	Purple
Z01-2-3-18	3106.267	0	PL	Purple
Z01-2-3-19	3084.971	0	PL	Purple
Z01-2-3-20	3106.267	0	PL	Purple
Z01-2-3-21	3106.267	0	PL	Purple
Z01-2-3-22	3106.267	0	PL	Purple
Z01-2-3-23	3651.568	0	PL	Purple
Z01-2-3-24	3651.568	0	PL	Purple
Z01-2-3-25	3651.568	0	PL	Purple
Z01-2-3-26	3651.568	0	PL	Purple
Z01-91-4-1	163.015	0	PL	Green
Z01-91-4-2	353.184	0	PL	Green
Z01-91-4-3	556.348	0	PL	Green
Z01-91-4-4	396.898	0	PL	Green
Z01-91-4-5	664.614	0	PL	Green
Z01-91-4-6	399.65	0	PL	Green
Z01-91-4-7	533.991	0	PL	Green
Z01-91-4-8	313.609	0	PL	Green
Z01-91-4-9	2933.401	0	PL	Green
Z01-91-4-10	2933.401	0	PL	Green
Z01-91-4-11	2763.386	0	PL	Green
Z01-91-4-12	2933.401	0	PL	Green
Z01-91-4-13	2933.401	0	PL	Green
Z01-91-4-14	2933.401	0	PL	Green
Z01-91-4-15	2933.401	0	PL	Green
Z01-91-4-16	2933.401	0	PL	Green
Z01-91-4-17	2763.386	0	PL	Green
Z01-91-4-18	2763.386	0	PL	Green
Z01-91-4-19	3086.374	0	PL	Green
Z01-91-4-20	3086.374	0	PL	Green
Z01-91-4-21	3086.374	0	PL	Green
Z01-91-4-22	3086.374	0	PL	Green
Z01-91-4-23	3086.374	0	PL	Green
Z01-91-4-24	3086.374	0	PL	Green
Z01-91-4-25	2763.386	0	PL	Green
Z01-91-4-26	3086.374	0	PL	Green
Z01-91-5-1	976.229	0	PL	Green
Z01-91-5-2	1023.825	0	PL	Green
Z01-91-5-3	677.492	0	PL	Green
Z01-91-5-4	405.67	0	PL	Green
Z01-91-5-5	496.058	0	PL	Green
Z01-91-5-6	490.522	0	PL	Green
Z01-91-5-7	257.982	0	PL	Green
Z01-91-5-8	487.119	0	PL	Green

Z01-91-5-9	3970.178	0	PL	Green
Z01-91-5-10	3949.541	0	PL	Green
Z01-91-5-11	3949.541	0	PL	Green
Z01-91-5-12	3970.178	0	PL	Green
Z01-91-5-13	3949.541	0	PL	Green
Z01-91-5-14	3154.954	0	PL	Green
Z01-91-5-15	3970.178	0	PL	Green
Z01-91-5-16	3154.954	0	PL	Green
Z01-91-5-17	3154.954	0	PL	Green
Z01-91-5-18	3154.954	0	PL	Green
Z01-91-5-19	3949.541	0	PL	Green
Z01-91-5-20	3949.541	0	PL	Green
Z01-91-5-21	3154.954	0	PL	Green
Z01-91-5-22	3154.954	0	PL	Green
Z01-91-5-23	3949.541	0	PL	Green
Z01-91-5-24	3949.541	0	PL	Green
Z01-91-5-25	3970.178	0	PL	Green
Z01-91-5-26	3970.178	0	PL	Green
Z01-95-1-1	352.869	0	PL	Green
Z01-95-1-2	281.771	0	PL	Green
Z01-95-1-3	68.901	0	PL	Green
Z01-95-1-4	151.103	0	PL	Green
Z01-95-1-5	125.635	0	PL	Green
Z01-95-1-6	6169.571	0	PL	Green
Z01-95-1-7	5323.435	0	PL	Green
Z01-95-1-8	1917.122	0	PL	Green
Z01-95-1-9	2900.221	0	PL	Green
Z01-95-1-10	2900.221	0	PL	Green
Z01-95-1-11	7624.808	0	PL	Green
Z01-95-1-12	2900.221	0	PL	Green
Z01-95-1-13	8788.906	0	PL	Green
Z01-95-1-14	2900.221	0	PL	Green
Z01-95-1-15	5779.894	0	PL	Green
Z01-95-1-16	978.562	0	PL	Green
Z01-95-1-17	2900.221	0	PL	Green
Z01-95-1-18	11237.795	0	PL	Green
Z01-95-1-19	3710.256	0	PL	Green
Z01-95-1-20	8634.845	0	PL	Green
Z01-95-1-21	2900.221	0	PL	Green
Z01-95-1-22	3154.954	0	PL	Green
Z01-95-1-23	1820.953	0	PL	Green
Z01-95-1-24	3154.954	0	PL	Green
Z01-95-1-25	2900.221	0	PL	Green
Z01-95-1-26	976.089	0	PL	Green

Z01-95-2-1	216.698	0	PL	Green
Z01-95-2-2	112.694	0	PL	Green
Z01-95-2-3	79.218	0	PL	Green
Z01-95-2-4	92.72	0	PL	Green
Z01-95-2-5	41.153	0	PL	Green
Z01-95-2-8	72.111	0	PL	Green
Z01-95-2-9	2717.772	0	PL	Green
Z01-95-2-10	2900.221	0	PL	Green
Z01-95-2-11	2900.221	0	PL	Green
Z01-95-2-12	2900.221	0	PL	Green
Z01-95-2-13	2900.221	0	PL	Green
Z01-95-2-14	2503.204	0	PL	Green
Z01-95-2-15	2503.204	0	PL	Green
Z01-95-2-16	2503.204	0	PL	Green
Z01-95-2-17	2900.221	0	PL	Green
Z01-95-2-18	2900.221	0	PL	Green
Z01-95-2-19	2717.772	0	PL	Green
Z01-95-2-20	2717.772	0	PL	Green
Z01-95-2-21	2717.772	0	PL	Green
Z01-95-2-22	2717.772	0	PL	Green
Z01-95-2-23	2717.772	0	PL	Green
Z01-95-2-24	2717.772	0	PL	Green
Z01-95-2-25	2503.204	0	PL	Green
Z01-95-2-26	2503.204	0	PL	Green

Table S11. Chemotype and leaf color identification of individuals in F_5 populations of Z01.

Sample Name	Perillene	Perillaldehyde	Chemotype	Leaf color
Z01-1-91-4-2	114.54	0	PL	Green
Z01-1-91-4-7	147.072	0	PL	Green
Z01-1-91-4-16	165.284	0	PL	Green
Z01-1-91-4-24	127.651	0	PL	Green
Z01-1-91-4-27	135.484	0	PL	Green
Z01-1-91-5-1	116.173	0	PL	Green
Z01-1-91-5-6	74.208	0	PL	Green
Z01-1-91-5-10	248.798	0	PL	Green
Z01-1-91-5-12	179.742	0	PL	Green
Z01-1-91-5-13	478.981	0	PL	Green
Z01-1-95-2-1	260.367	0	PL	Green
Z01-1-95-2-14	280.237	0	PL	Green
Z01-1-95-2-17	109.344	0	PL	Green
Z01-1-95-2-19	69.662	0	PL	Green
Z01-1-95-2-27	185.227	0	PL	Green
Z01-1-2-3-5	140.344	0	PL	Purple
Z01-1-2-3-6	159.746	0	PL	Purple
Z01-1-2-3-20	168.209	0	PL	Purple
Z01-1-2-3-22	226.269	0	PL	Purple
Z01-1-2-3-27	139.897	0	PL	Purple

Table S12. Chromosomal distribution of genes of leaf color and chemotypes.

Chromosome	Position	Locus	Gene type
Pfg10	48.85773	PFCHS2(Pfg10_26810)	Leaf color
Pfg13	13.40535	PFCHS2(Pfg13_11980)	Leaf color
Pfg01	15.94537	Pf3-GT(Pfg01_12990)	Leaf color
Pfg01	55.2359	Pf3-GT(Pfg01_35760)	Leaf color
Pfg04	62.62207	Pf5-GT1(Pfg04_31530)	Leaf color
Pfg04	62.62224	Pf5-GT1(Pfg04_31540)	Leaf color
Pfg06	8.695943	Pf5-GT1(Pfg06_08210)	Leaf color
Pfg07	36.72403	PfACT(Pfg07_29360)	Leaf color
Pfg19	19.78212	PfACT(Pfg19_15480)	Leaf color
Pfg14	15.1383	PfANS(Pfg14_13790)	Leaf color
Pfg18	15.20988	PfANS(Pfg18_14070)	Leaf color
Pfg11	47.33201	PfC4H1(Pfg11_34950)	Leaf color
Pfg12	9.906059	PfC4H1(Pfg12_11510)	Leaf color
Pfg02	22.02189	PfCHI(Pfg02_12460)	Leaf color
Pfg12	15.59907	PfCHI(Pfg12_16170)	Leaf color
Pfg10	48.85773	PfCHS1(Pfg10_26810)	Leaf color
Pfg13	13.40535	PfCHS1(Pfg13_11980)	Leaf color
Pfg02	77.19611	PfDFR(Pfg02_66420)	Leaf color
Pfg12	0.353848	PfDFR(Pfg12_00480)	Leaf color
Pfg07	8.546597	PfF3H1(Pfg07_05480)	Leaf color
Pfg19	42.7195	PfF3H1(Pfg19_27030)	Leaf color
Pfg07	8.546597	PfF3H2(Pfg07_05480)	Leaf color
Pfg19	42.7195	PfF3H2(Pfg19_27030)	Leaf color
Pfg03	17.34232	PfF3H(Pfg03_11050)	Leaf color
Pfg09	12.97406	PfF3H(Pfg09_08970)	Leaf color
Pfg08	11.60415	PfMYB113b(Pfg08_10680)	Leaf color
Pfg16	12.34475	PfMYB113b(Pfg16_10470)	Leaf color
Pfg05	23.74429	CYP71AT146(Pfg05_12650)	Chemotype
Pfg05	23.79247	CYP71AT146(Pfg05_12670)	Chemotype
Pfg05	23.82131	CYP71AT146(Pfg05_12680)	Chemotype
Pfg05	23.83728	CYP71AT146(Pfg05_12690)	Chemotype
Pfg05	23.88172	CYP71AT146(Pfg05_12710)	Chemotype
Pfg05	25.88834	CYP71AT146(Pfg05_13190)	Chemotype
Pfg05	25.94264	CYP71AT146(Pfg05_13210)	Chemotype
Pfg05	25.95815	CYP71AT146(Pfg05_13220)	Chemotype
Pfg05	25.9753	CYP71AT146(Pfg05_13230)	Chemotype
Pfg05	25.99122	CYP71AT146(Pfg05_13240)	Chemotype
Pfg05	26.0356	CYP71AT146(Pfg05_13270)	Chemotype
Pfg08	29.4661	CYP71D174(Pfg08_21820)	Chemotype
Pfg16	48.78393	CYP71D174(Pfg16_29750)	Chemotype
Pfg05	23.42312	LC(Pfg05_12580)	Chemotype

Pfg05	25.54409	LC(Pfg05_13070)	Chemotype
Pfg05	27.71468	LC(Pfg05_13690)	Chemotype
Pfg05	27.71962	LC(Pfg05_13700)	Chemotype
Pfg03	1.996857	PTS-5526-MS(Pfg03_02100)	Chemotype
Pfg09	1.943727	PTS-5526-MS(Pfg09_01960)	Chemotype
Pfg08	24.57494	PcAKR(Pfg08_18560)	Chemotype
Pfg08	24.58457	PcAKR(Pfg08_18580)	Chemotype
Pfg08	24.58529	PcAKR(Pfg08_18590)	Chemotype
Pfg16	53.61003	PcAKR(Pfg16_32670)	Chemotype
Pfg16	53.61262	PcAKR(Pfg16_32680)	Chemotype
Pfg16	53.61908	PcAKR(Pfg16_32690)	Chemotype
Pfg08	7.262086	PcGeDH(Pfg08_07830)	Chemotype
Pfg16	7.876471	PcGeDH(Pfg16_07640)	Chemotype
Pfg15	43.83169	PcPT-ISPD2(Pfg15_27880)	Chemotype
Pfg03	68.61696	PcTps-C-GS(Pfg03_31470)	Chemotype
Pfg09	55.84501	PcTps-C-GS(Pfg09_29010)	Chemotype
Pfg03	68.61696	PcTps-EK-GS(Pfg03_31470)	Chemotype
Pfg09	55.84501	PcTps-EK-GS(Pfg09_29010)	Chemotype
Pfg03	68.61696	PcTps-PK-GS(Pfg03_31470)	Chemotype
Pfg09	55.84501	PcTps-PK-GS(Pfg09_29010)	Chemotype
Pfg08	24.57494	PfAKR(Pfg08_18560)	Chemotype
Pfg08	24.58457	PfAKR(Pfg08_18580)	Chemotype
Pfg08	24.58529	PfAKR(Pfg08_18590)	Chemotype
Pfg16	53.61003	PfAKR(Pfg16_32670)	Chemotype
Pfg16	53.61262	PfAKR(Pfg16_32680)	Chemotype
Pfg16	53.61908	PfAKR(Pfg16_32690)	Chemotype
Pfg08	7.262086	PfGeDH(Pfg08_07830)	Chemotype
Pfg16	7.876471	PfGeDH(Pfg16_07640)	Chemotype
Pfg03	68.61696	PfTps-PL-GS(Pfg03_31470)	Chemotype
Pfg09	55.84501	PfTps-PL-GS(Pfg09_29010)	Chemotype
Pfg03	68.61696	PhTps-5073G-GS(Pfg03_31470)	Chemotype
Pfg09	55.84501	PhTps-5073G-GS(Pfg09_29010)	Chemotype
Pfg08	24.57494	PsAKR(Pfg08_18560)	Chemotype
Pfg08	24.58457	PsAKR(Pfg08_18580)	Chemotype
Pfg08	24.58529	PsAKR(Pfg08_18590)	Chemotype
Pfg16	53.61003	PsAKR(Pfg16_32670)	Chemotype
Pfg16	53.61262	PsAKR(Pfg16_32680)	Chemotype
Pfg16	53.61908	PsAKR(Pfg16_32690)	Chemotype
Pfg08	7.262086	PsGeDH(Pfg08_07830)	Chemotype
Pfg16	7.876471	PsGeDH(Pfg16_07640)	Chemotype
Pfg03	68.61696	PsTps-5031G-GS(Pfg03_31470)	Chemotype
Pfg09	55.84501	PsTps-5031G-GS(Pfg09_29010)	Chemotype
